# Aqua­bis(4-methyl­benzoato)-κ*O*;κ^2^ *O*,*O*′-bis­(pyridine-κ*N*)nickel(II)

**DOI:** 10.1107/S160053680800634X

**Published:** 2008-03-14

**Authors:** Wen-Dong Song, Hao Wang, Li-Li Ji

**Affiliations:** a College of Science, Guang Dong Ocean University, Zhanjiang 524088, People’s Republic of China

## Abstract

In the title mononuclear complex, [Ni(C_8_H_7_O_2_)_2_(C_5_H_5_N)_2_(H_2_O)], the Ni^II^ atom is in a distorted octa­hedral arrangement, coordinated by three carboxylate O atoms from one bidentate 4-methyl­benzoate ligand and one monodentate 4-methyl­benzoate ligand, two N atoms from pyridine ligands, axially positioned, and a water mol­ecule. The equatorially positioned water mol­ecule and uncoordinated carb­oxylate O atom form an intra­molecular hydrogen bond. An inter­molecular O—H⋯O hydrogen bond between the coordinated water mol­ecule and carboxylate O atom of the 4-methyl­benzoate ligand forms infinite chains along the *b* axis. These chains are connected by C—H⋯π inter­actions.

## Related literature

For related literature, see: Song *et al.* (2007 ▶).
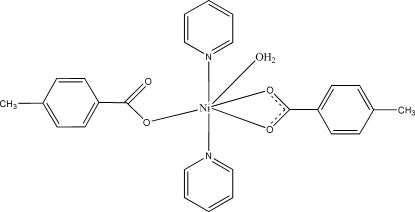


## Experimental

### 

#### Crystal data

[Ni(C_8_H_7_O_2_)_2_(C_5_H_5_N)_2_(H_2_O)]
*M* *_r_* = 505.20
Monoclinic, 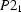

*a* = 13.6181 (1) Å
*b* = 5.9526 (1) Å
*c* = 15.1380 (2) Å
β = 107.215 (1)°
*V* = 1172.16 (3) Å^3^
*Z* = 2
Mo *K*α radiation
μ = 0.87 mm^−1^
*T* = 296 (2) K
0.26 × 0.23 × 0.20 mm

#### Data collection

Bruker APEXII area-detector diffractometer
Absorption correction: multi-scan (*SADABS*; Sheldrick, 1996 ▶) *T*_min_ = 0.806, *T*_max_ = 0.846
11325 measured reflections
5102 independent reflections
4798 reflections with *I* > 2σ(*I*)
*R*_int_ = 0.023

#### Refinement

*R*[*F* ^2^ > 2σ(*F* ^2^)] = 0.028
*wR*(*F* ^2^) = 0.065
*S* = 1.03
5102 reflections
315 parameters
4 restraints
H atoms treated by a mixture of independent and constrained refinement
Δρ_max_ = 0.25 e Å^−3^
Δρ_min_ = −0.28 e Å^−3^
Absolute structure: Flack (1983 ▶)
Flack parameter: 0.00

### 

Data collection: *APEX2* (Bruker, 2004 ▶); cell refinement: *SAINT* (Bruker, 2004 ▶); data reduction: *SAINT*; program(s) used to solve structure: *SHELXS97* (Sheldrick, 2008 ▶); program(s) used to refine structure: *SHELXL97* (Sheldrick, 2008 ▶); molecular graphics: *XP* in *SHELXTL* (Sheldrick, 2008 ▶); software used to prepare material for publication: *SHELXL97* and *XP* in *SHELXTL*.

## Supplementary Material

Crystal structure: contains datablocks I, global. DOI: 10.1107/S160053680800634X/kp2154sup1.cif

Structure factors: contains datablocks I. DOI: 10.1107/S160053680800634X/kp2154Isup2.hkl

Additional supplementary materials:  crystallographic information; 3D view; checkCIF report

## Figures and Tables

**Table:** 

N1—Ni1	2.0941 (17)
N2—Ni1	2.0981 (16)
Ni1—O3	2.0165 (14)
Ni1—O1*W*	2.0412 (15)
Ni1—O2	2.1107 (12)
Ni1—O1	2.1710 (15)

**Table:** 

O3—Ni1—O1*W*	94.41 (6)
O3—Ni1—N1	86.71 (6)
O1*W*—Ni1—N1	89.53 (6)
O3—Ni1—N2	89.22 (6)
O1*W*—Ni1—N2	92.99 (6)
N1—Ni1—N2	175.36 (7)
O3—Ni1—O2	165.26 (7)
O1*W*—Ni1—O2	99.83 (8)
N1—Ni1—O2	89.57 (6)
N2—Ni1—O2	93.83 (6)
O3—Ni1—O1	104.00 (6)
O1*W*—Ni1—O1	161.59 (5)
N1—Ni1—O1	91.67 (6)
N2—Ni1—O1	87.17 (6)
O2—Ni1—O1	61.82 (7)

**Table 2:** Hydrogen-bond geometry (Å, °)

*D*—H⋯*A*	*D*—H	H⋯*A*	*D*⋯*A*	*D*—H⋯*A*
O1*W*—H2*W*⋯O4	0.819 (9)	1.834 (13)	2.587 (2)	152 (2)
O1*W*—H1*W*⋯O1^i^	0.809 (9)	1.957 (12)	2.739 (2)	162 (2)

Symmetry code: (i) 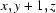.

**Fig. 1.**
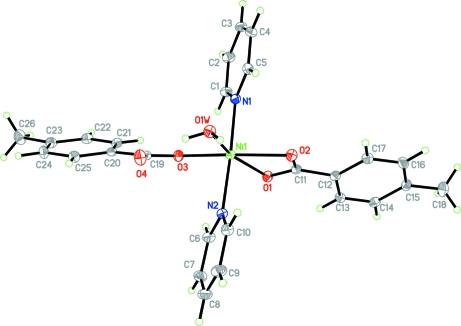
The structure of (I) showing the atomic numbering scheme and octahedral coordination of NiII. Non-H atoms are shown with the 30% probability displacement ellipsoids.

**Fig. 2.**
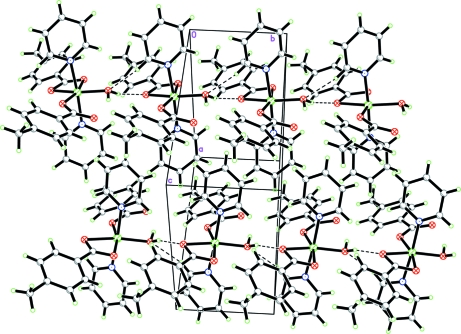
The crystal packing of (I). The intermolecluar hydrogen bonds are shown as dashed lines.

## supplementary crystallographic information

### Comment

In the structural investigation of 4-methylbenzoate complexes, it has been found that the 4-methylbenzoic acid functions as a multidentate ligand [Song *et al.* (2007)] with versatile binding and coordination modes. In this paper, we report the crystal structure of the title compound, (I), a new Ni complex obtained by the reaction of 4-methylbenzoic acid, pyridine and nickel chloride in alkaline aqueous solution.

The Ni^II^ atom exhibits a disordered octahedral environment (Fig. 1, Table 1) defined by three carboxyl O atoms from one bidentate 4-methylbenzoate ligand and one monodentate 4-methylbenzoate ligand, two N atoms from two pyridine ligands and a water molecule. The intermolecular O—H···O hydrogen bond (Table 2, Fig. 2) between the coordinated water molecule and carboxy O atom of 4-methylbenzoate ligand generates a chain along the axis b. The intermolecular hydrogen bond C1—H1···O also involves water molecule [3.339 (3) %A, 145%]. An intramolecular hydrogen bond connects the coordinated water molecule and uncoordinated oxygen atom O4 (Table 2). C—H···π interactions connect hydrogen bonded chains: C_3_– H_3_···C_g_ (C_12_→ C_17_, symmetry code: -2 - *x*, 1/2 + *y*, 2 - *z*) of 3.482 (2) %A; 132%, and C_14_– H14···C_g_(C_12_→ C_17_, symmetry code: 1 - *x*, -1/2 + *y*, 2 - *z*) of 3.603 (2) %A; 134%; C_22_– H22···C_g_(C_20_→ C_25_, symmetry code: 2 - *x*, -1/2 + *y*, 1 - *z*) of 3.504 (2) %A; 133%.

### Experimental

A mixture of nickel chloride (1 mmol), 4-methylbenzoic acid (1 mmol), pyridine(1 mmol), NaOH (1.5 mmol) and H_2_O (12 ml) were placed into a 23 ml Teflon reactor, which was heated to 433 K for three days and then cooled to room temperature at a rate of 10 K h^-1^. The crystals obtained were washed with water and dryed in air.

### Refinement

Carbon-bound H atoms were placed at calculated positions and were treated as riding on the parent C atoms with C—H = 0.93 Å for aromatic rings, C—H = 0.96 Å for methyl group, and with *U*_iso_(H) = 1.2 *U*_eq_(C). Water H atoms were tentatively located in difference Fourier maps and were refined with distance restraints of O–H = 0.82 Å and H···H = 1.29 Å, each within a standard deviation of 0.01 Å; and with *U*_iso_(H) = 1.5 *U*_eq_(O).

### Crystal data

**Table:**

[Ni(C_8_H_7_O_2_)_2_(C_5_H_5_N)_2_(H_2_O)]	*F*_000_ = 528
*M**_r_* = 505.20	*D*_x_ = 1.431 Mg m^−^^3^
Monoclinic, *P*2_1_	Mo *K*α radiation λ = 0.71073 Å
Hall symbol: P 2yb	Cell parameters from 4520 reflections
*a* = 13.61810 (10) Å	θ = 1.4–28º
*b* = 5.95260 (10) Å	µ = 0.87 mm^−^^1^
*c* = 15.1380 (2) Å	*T* = 296 (2) K
β = 107.2150 (10)º	Block, blue
*V* = 1172.16 (3) Å^3^	0.26 × 0.23 × 0.20 mm
*Z* = 2	

### Data collection

**Table:**

Bruker APEXII area-detector diffractometer	5102 independent reflections
Radiation source: fine-focus sealed tube	4798 reflections with *I* > 2σ(*I*)
Monochromator: graphite	*R*_int_ = 0.023
*T* = 296(2) K	θ_max_ = 27.5º
φ and ω scans	θ_min_ = 1.6º
Absorption correction: multi-scan(SADABS; Sheldrick, 1996)	*h* = −17→17
*T*_min_ = 0.806, *T*_max_ = 0.846	*k* = −7→7
11325 measured reflections	*l* = −19→17

### Refinement

**Table:**

Refinement on *F*^2^	Hydrogen site location: inferred from neighbouring sites
Least-squares matrix: full	H atoms treated by a mixture of independent and constrained refinement
*R*[*F*^2^ > 2σ(*F*^2^)] = 0.028	*w* = 1/[σ^2^(*F*_o_^2^) + (0.0307*P*)^2^ + 0.1736*P*] where *P* = (*F*_o_^2^ + 2*F*_c_^2^)/3
*wR*(*F*^2^) = 0.065	(Δ/σ)_max_ = 0.001
*S* = 1.04	Δρ_max_ = 0.25 e Å^−^^3^
5102 reflections	Δρ_min_ = −0.28 e Å^−^^3^
315 parameters	Extinction correction: none
4 restraints	Absolute structure: Flack (1983)
Primary atom site location: structure-invariant direct methods	Flack parameter: 0.00
Secondary atom site location: difference Fourier map	

### Special details

**Table:**

Geometry. All e.s.d.'s (except the e.s.d. in the dihedral angle between two l.s. planes) are estimated using the full covariance matrix. The cell e.s.d.'s are taken into account individually in the estimation of e.s.d.'s in distances, angles and torsion angles; correlations between e.s.d.'s in cell parameters are only used when they are defined by crystal symmetry. An approximate (isotropic) treatment of cell e.s.d.'s is used for estimating e.s.d.'s involving l.s. planes.
Refinement. Refinement of *F*^2^ against ALL reflections. The weighted *R*-factor *wR* and goodness of fit *S* are based on *F*^2^, conventional *R*-factors *R* are based on *F*, with *F* set to zero for negative *F*^2^. The threshold expression of *F*^2^ > σ(*F*^2^) is used only for calculating *R*-factors(gt) *etc*. and is not relevant to the choice of reflections for refinement. *R*-factors based on *F*^2^ are statistically about twice as large as those based on *F*, and *R*- factors based on ALL data will be even larger.

### Fractional atomic coordinates and isotropic or equivalent isotropic displacement parameters (Å^2^)

**Table:**

	*x*	*y*	*z*	*U*_iso_*/*U*_eq_	
C1	0.95095 (15)	0.1169 (4)	0.82231 (15)	0.0247 (4)	
H1	0.9142	0.0108	0.7803	0.030*	
C2	1.05343 (16)	0.0757 (4)	0.86802 (16)	0.0296 (5)	
H2	1.0845	−0.0563	0.8571	0.036*	
C3	1.10915 (16)	0.2322 (4)	0.92999 (16)	0.0305 (5)	
H3	1.1782	0.2083	0.9613	0.037*	
C4	1.06002 (16)	0.4243 (4)	0.94418 (15)	0.0292 (5)	
H4	1.0954	0.5331	0.9855	0.035*	
C5	0.95739 (16)	0.4540 (4)	0.89642 (15)	0.0261 (5)	
H5	0.9250	0.5847	0.9067	0.031*	
C6	0.55162 (16)	0.2440 (4)	0.62678 (16)	0.0272 (5)	
H6	0.5903	0.1158	0.6259	0.033*	
C7	0.45288 (17)	0.2553 (4)	0.56751 (17)	0.0322 (5)	
H7	0.4258	0.1367	0.5278	0.039*	
C8	0.39535 (17)	0.4435 (4)	0.56807 (18)	0.0352 (6)	
H8	0.3286	0.4545	0.5288	0.042*	
C9	0.43788 (17)	0.6170 (4)	0.62781 (17)	0.0345 (5)	
H9	0.4004	0.7468	0.6292	0.041*	
C10	0.53759 (16)	0.5937 (4)	0.68568 (15)	0.0273 (5)	
H10	0.5661	0.7104	0.7260	0.033*	
C11	0.69445 (14)	0.1687 (3)	0.88686 (14)	0.0200 (4)	
C12	0.66663 (15)	0.0548 (4)	0.96375 (14)	0.0214 (4)	
C13	0.62152 (14)	−0.1580 (4)	0.95154 (13)	0.0227 (4)	
H13	0.6066	−0.2280	0.8941	0.027*	
C14	0.59873 (15)	−0.2661 (4)	1.02416 (15)	0.0257 (5)	
H14	0.5669	−0.4059	1.0143	0.031*	
C15	0.62267 (15)	−0.1690 (4)	1.11155 (14)	0.0263 (5)	
C16	0.66774 (16)	0.0436 (4)	1.12359 (15)	0.0289 (5)	
H16	0.6843	0.1115	1.1815	0.035*	
C17	0.68834 (16)	0.1557 (4)	1.05043 (15)	0.0252 (4)	
H17	0.7168	0.2990	1.0594	0.030*	
C18	0.6011 (2)	−0.2912 (5)	1.19129 (17)	0.0405 (6)	
H18A	0.6522	−0.2515	1.2479	0.061*	
H18B	0.6030	−0.4503	1.1816	0.061*	
H18C	0.5343	−0.2494	1.1950	0.061*	
C19	0.78596 (14)	0.4041 (4)	0.58580 (14)	0.0216 (5)	
C20	0.82429 (14)	0.3106 (3)	0.50984 (14)	0.0209 (5)	
C21	0.87038 (15)	0.0988 (4)	0.51907 (15)	0.0237 (4)	
H21	0.8747	0.0125	0.5713	0.028*	
C22	0.90959 (16)	0.0170 (4)	0.45091 (15)	0.0258 (5)	
H22	0.9409	−0.1235	0.4584	0.031*	
C23	0.90311 (16)	0.1405 (4)	0.37128 (15)	0.0285 (5)	
C24	0.85529 (15)	0.3503 (5)	0.36190 (13)	0.0294 (4)	
H24	0.8491	0.4350	0.3089	0.035*	
C25	0.81695 (16)	0.4341 (4)	0.43050 (15)	0.0258 (5)	
H25	0.7859	0.5748	0.4232	0.031*	
C26	0.9479 (2)	0.0517 (5)	0.29837 (16)	0.0423 (6)	
H26A	0.8973	0.0607	0.2389	0.063*	
H26B	0.9682	−0.1020	0.3118	0.063*	
H26C	1.0068	0.1398	0.2980	0.063*	
N1	0.90261 (12)	0.3038 (3)	0.83626 (12)	0.0219 (4)	
N2	0.59440 (12)	0.4102 (3)	0.68585 (12)	0.0213 (4)	
Ni1	0.748180 (17)	0.36693 (5)	0.764977 (16)	0.01841 (7)	
O1	0.69972 (10)	0.0586 (2)	0.81637 (9)	0.0226 (3)	
O2	0.71436 (10)	0.3781 (4)	0.89227 (9)	0.0241 (3)	
O3	0.78210 (11)	0.2707 (2)	0.64966 (10)	0.0232 (3)	
O4	0.76152 (12)	0.6085 (3)	0.58101 (10)	0.0310 (4)	
O1W	0.78338 (11)	0.6967 (2)	0.75308 (10)	0.0245 (3)	
H2W	0.7754 (18)	0.715 (4)	0.6978 (7)	0.037*	
H1W	0.7470 (16)	0.789 (3)	0.7669 (14)	0.037*	

### Atomic displacement parameters (Å^2^)

**Table:**

	*U*^11^	*U*^22^	*U*^33^	*U*^12^	*U*^13^	*U*^23^
C1	0.0227 (10)	0.0198 (10)	0.0305 (11)	−0.0009 (9)	0.0060 (9)	−0.0009 (9)
C2	0.0248 (11)	0.0261 (12)	0.0374 (13)	0.0035 (10)	0.0083 (9)	0.0022 (10)
C3	0.0191 (10)	0.0396 (14)	0.0307 (12)	−0.0009 (10)	0.0041 (9)	0.0063 (11)
C4	0.0276 (10)	0.0332 (15)	0.0241 (11)	−0.0077 (9)	0.0036 (9)	−0.0048 (9)
C5	0.0287 (11)	0.0266 (11)	0.0232 (11)	0.0003 (9)	0.0079 (9)	−0.0019 (8)
C6	0.0235 (10)	0.0251 (12)	0.0319 (12)	0.0003 (9)	0.0066 (9)	−0.0033 (10)
C7	0.0285 (12)	0.0341 (13)	0.0308 (13)	−0.0050 (11)	0.0038 (10)	−0.0029 (10)
C8	0.0218 (10)	0.0407 (14)	0.0387 (14)	−0.0009 (10)	0.0021 (10)	0.0098 (10)
C9	0.0245 (11)	0.0316 (13)	0.0466 (15)	0.0077 (10)	0.0092 (10)	0.0048 (11)
C10	0.0279 (11)	0.0249 (11)	0.0293 (11)	0.0008 (9)	0.0087 (9)	−0.0014 (9)
C11	0.0161 (9)	0.0205 (11)	0.0235 (10)	0.0041 (8)	0.0061 (8)	0.0006 (8)
C12	0.0189 (9)	0.0206 (11)	0.0253 (10)	0.0048 (8)	0.0075 (8)	0.0004 (9)
C13	0.0230 (8)	0.0204 (12)	0.0239 (9)	0.0016 (9)	0.0056 (7)	−0.0027 (10)
C14	0.0229 (10)	0.0232 (11)	0.0316 (12)	−0.0011 (9)	0.0089 (9)	−0.0001 (9)
C15	0.0252 (9)	0.0285 (15)	0.0271 (10)	0.0042 (9)	0.0108 (8)	0.0047 (10)
C16	0.0294 (11)	0.0341 (13)	0.0224 (11)	0.0022 (10)	0.0067 (9)	−0.0055 (10)
C17	0.0251 (10)	0.0218 (11)	0.0284 (11)	0.0000 (9)	0.0076 (9)	−0.0040 (9)
C18	0.0501 (15)	0.0426 (15)	0.0337 (14)	0.0007 (13)	0.0202 (12)	0.0064 (12)
C19	0.0159 (8)	0.0243 (15)	0.0230 (10)	0.0013 (9)	0.0032 (7)	−0.0002 (9)
C20	0.0156 (8)	0.0236 (12)	0.0222 (10)	−0.0017 (7)	0.0037 (7)	−0.0029 (8)
C21	0.0202 (9)	0.0229 (11)	0.0275 (11)	0.0000 (9)	0.0065 (8)	0.0020 (9)
C22	0.0231 (10)	0.0220 (11)	0.0327 (12)	0.0028 (9)	0.0089 (9)	−0.0038 (10)
C23	0.0258 (10)	0.0332 (13)	0.0272 (11)	−0.0001 (9)	0.0090 (9)	−0.0065 (10)
C24	0.0350 (10)	0.0332 (12)	0.0216 (9)	0.0018 (13)	0.0106 (8)	0.0035 (13)
C25	0.0258 (10)	0.0223 (11)	0.0290 (11)	0.0034 (8)	0.0076 (9)	0.0018 (8)
C26	0.0485 (15)	0.0510 (17)	0.0313 (13)	0.0134 (13)	0.0177 (11)	−0.0049 (13)
N1	0.0202 (8)	0.0233 (10)	0.0221 (8)	−0.0003 (6)	0.0061 (7)	0.0014 (7)
N2	0.0198 (7)	0.0215 (12)	0.0228 (8)	−0.0004 (7)	0.0066 (6)	−0.0004 (7)
Ni1	0.01837 (11)	0.01668 (11)	0.02025 (11)	0.00073 (12)	0.00583 (8)	−0.00085 (14)
O1	0.0258 (7)	0.0196 (7)	0.0244 (7)	0.0010 (6)	0.0103 (6)	−0.0028 (6)
O2	0.0285 (6)	0.0193 (7)	0.0264 (7)	−0.0009 (9)	0.0110 (5)	−0.0020 (9)
O3	0.0260 (7)	0.0231 (7)	0.0221 (8)	0.0015 (6)	0.0097 (6)	−0.0011 (6)
O4	0.0437 (9)	0.0232 (8)	0.0288 (8)	0.0098 (7)	0.0151 (7)	0.0014 (7)
O1W	0.0283 (7)	0.0186 (8)	0.0274 (8)	0.0013 (6)	0.0097 (6)	−0.0031 (7)

### Geometric parameters (Å, °)

**Table:**

C1—N1	1.341 (3)	C15—C18	1.511 (3)
C1—C2	1.384 (3)	C16—C17	1.391 (3)
C1—H1	0.9300	C16—H16	0.9300
C2—C3	1.379 (3)	C17—H17	0.9300
C2—H2	0.9300	C18—H18A	0.9600
C3—C4	1.373 (3)	C18—H18B	0.9600
C3—H3	0.9300	C18—H18C	0.9600
C4—C5	1.382 (3)	C19—O4	1.258 (3)
C4—H4	0.9300	C19—O3	1.264 (3)
C5—N1	1.335 (3)	C19—C20	1.503 (3)
C5—H5	0.9300	C20—C25	1.386 (3)
C6—N2	1.345 (3)	C20—C21	1.396 (3)
C6—C7	1.380 (3)	C21—C22	1.383 (3)
C6—H6	0.9300	C21—H21	0.9300
C7—C8	1.369 (3)	C22—C23	1.393 (3)
C7—H7	0.9300	C22—H22	0.9300
C8—C9	1.382 (4)	C23—C24	1.396 (4)
C8—H8	0.9300	C23—C26	1.506 (3)
C9—C10	1.388 (3)	C24—C25	1.386 (3)
C9—H9	0.9300	C24—H24	0.9300
C10—N2	1.338 (3)	C25—H25	0.9300
C10—H10	0.9300	C26—H26A	0.9600
C11—O1	1.272 (2)	C26—H26B	0.9600
C11—O2	1.273 (3)	C26—H26C	0.9600
C11—C12	1.490 (3)	N1—Ni1	2.0941 (17)
C12—C17	1.393 (3)	N2—Ni1	2.0981 (16)
C12—C13	1.396 (3)	Ni1—O3	2.0165 (14)
C13—C14	1.386 (3)	Ni1—O1W	2.0412 (15)
C13—H13	0.9300	Ni1—O2	2.1107 (12)
C14—C15	1.391 (3)	Ni1—O1	2.1710 (15)
C14—H14	0.9300	O1W—H2W	0.819 (9)
C15—C16	1.395 (3)	O1W—H1W	0.809 (9)
			
N1—C1—C2	122.5 (2)	H18B—C18—H18C	109.5
N1—C1—H1	118.7	O4—C19—O3	125.6 (2)
C2—C1—H1	118.7	O4—C19—C20	117.39 (19)
C3—C2—C1	119.4 (2)	O3—C19—C20	117.1 (2)
C3—C2—H2	120.3	C25—C20—C21	118.70 (19)
C1—C2—H2	120.3	C25—C20—C19	120.93 (19)
C4—C3—C2	118.2 (2)	C21—C20—C19	120.35 (18)
C4—C3—H3	120.9	C22—C21—C20	120.3 (2)
C2—C3—H3	120.9	C22—C21—H21	119.8
C3—C4—C5	119.3 (2)	C20—C21—H21	119.8
C3—C4—H4	120.4	C21—C22—C23	121.3 (2)
C5—C4—H4	120.4	C21—C22—H22	119.3
N1—C5—C4	123.1 (2)	C23—C22—H22	119.3
N1—C5—H5	118.5	C22—C23—C24	117.94 (19)
C4—C5—H5	118.5	C22—C23—C26	120.9 (2)
N2—C6—C7	122.9 (2)	C24—C23—C26	121.2 (2)
N2—C6—H6	118.5	C25—C24—C23	120.9 (2)
C7—C6—H6	118.5	C25—C24—H24	119.5
C8—C7—C6	119.0 (2)	C23—C24—H24	119.5
C8—C7—H7	120.5	C20—C25—C24	120.8 (2)
C6—C7—H7	120.5	C20—C25—H25	119.6
C7—C8—C9	119.1 (2)	C24—C25—H25	119.6
C7—C8—H8	120.5	C23—C26—H26A	109.5
C9—C8—H8	120.5	C23—C26—H26B	109.5
C8—C9—C10	118.7 (2)	H26A—C26—H26B	109.5
C8—C9—H9	120.6	C23—C26—H26C	109.5
C10—C9—H9	120.6	H26A—C26—H26C	109.5
N2—C10—C9	122.7 (2)	H26B—C26—H26C	109.5
N2—C10—H10	118.7	C5—N1—C1	117.51 (18)
C9—C10—H10	118.7	C5—N1—Ni1	120.29 (14)
O1—C11—O2	119.64 (18)	C1—N1—Ni1	122.19 (14)
O1—C11—C12	120.77 (18)	C10—N2—C6	117.56 (17)
O2—C11—C12	119.58 (17)	C10—N2—Ni1	125.46 (14)
C17—C12—C13	118.57 (19)	C6—N2—Ni1	116.87 (14)
C17—C12—C11	120.52 (19)	O3—Ni1—O1W	94.41 (6)
C13—C12—C11	120.87 (18)	O3—Ni1—N1	86.71 (6)
C14—C13—C12	120.61 (19)	O1W—Ni1—N1	89.53 (6)
C14—C13—H13	119.7	O3—Ni1—N2	89.22 (6)
C12—C13—H13	119.7	O1W—Ni1—N2	92.99 (6)
C13—C14—C15	121.1 (2)	N1—Ni1—N2	175.36 (7)
C13—C14—H14	119.4	O3—Ni1—O2	165.26 (7)
C15—C14—H14	119.4	O1W—Ni1—O2	99.83 (8)
C14—C15—C16	118.13 (19)	N1—Ni1—O2	89.57 (6)
C14—C15—C18	120.9 (2)	N2—Ni1—O2	93.83 (6)
C16—C15—C18	121.0 (2)	O3—Ni1—O1	104.00 (6)
C17—C16—C15	121.1 (2)	O1W—Ni1—O1	161.59 (5)
C17—C16—H16	119.5	N1—Ni1—O1	91.67 (6)
C15—C16—H16	119.5	N2—Ni1—O1	87.17 (6)
C16—C17—C12	120.4 (2)	O2—Ni1—O1	61.82 (7)
C16—C17—H17	119.8	C11—O1—Ni1	87.92 (12)
C12—C17—H17	119.8	C11—O2—Ni1	90.59 (12)
C15—C18—H18A	109.5	C19—O3—Ni1	123.76 (14)
C15—C18—H18B	109.5	Ni1—O1W—H2W	104.8 (19)
H18A—C18—H18B	109.5	Ni1—O1W—H1W	117.0 (18)
C15—C18—H18C	109.5	H2W—O1W—H1W	105.6 (15)
H18A—C18—H18C	109.5		

### Hydrogen-bond geometry (Å, °)

**Table:**

*D*—H···*A*	*D*—H	H···*A*	*D*···*A*	*D*—H···*A*
O1W—H2W···O4	0.819 (9)	1.834 (13)	2.587 (2)	152 (2)
O1W—H1W···O1^i^	0.809 (9)	1.957 (12)	2.739 (2)	162 (2)

Symmetry codes: (i) *x*, *y*+1, *z*.
